# A Mendelian randomization study of testosterone and cognition in men

**DOI:** 10.1038/srep21306

**Published:** 2016-02-11

**Authors:** Jie V. Zhao, Tai Hing Lam, Chaoqiang Jiang, Stacey S. Cherny, Bin Liu, Kar Keung Cheng, Weisen Zhang, Gabriel M. Leung, C Mary Schooling

**Affiliations:** 1 School of Public Health, Li Ka Shing Faculty of Medicine, The University of Hong Kong, Hong Kong Special Administrative Region, Hong Kong, China; 2 Guangzhou Number 12 Hospital, Guangzhou, China; 3 Department of Psychiatry, Li Ka Shing Faculty of Medicine, The University of Hong Kong, Hong Kong SAR, China; 4 Department of Public Health and Epidemiology, University of Birmingham, Birmingham, United Kingdom; 5 CUNY School of Public Health and Health Policy, New York, New York, United States of America

## Abstract

Testosterone replacement for older men is increasingly common, with some observations suggesting a protective effect on cognitive function. We examined the association of endogenous testosterone with cognitive function among older men in a Mendelian randomization study using a separate-sample instrumental variable (SSIV) analysis estimator to minimize confounding and reverse causality. A genetic score predicting testosterone was developed in 289 young Chinese men from Hong Kong, based on selected testosterone-related single nucleotide polymorphisms (rs10046, rs1008805 and rs1256031). The association of genetically predicted testosterone with delayed 10-word recall score and Mini-Mental State Examination (MMSE) score was assessed at baseline and follow-up using generalized estimating equation among 4,212 older Chinese men from the Guangzhou Biobank Cohort Study. Predicted testosterone was not associated with delayed 10-word recall score (−0.02 per nmol/L testosterone, 95% confidence interval (CI) −0.06–0.02) or MMSE score (0.06, 95% CI −0.002–0.12). These estimates were similar after additional adjustment for age, education, smoking, use of alcohol, body mass index and the Framingham score. Our findings do not corroborate observed protective effects of testosterone on cognitive function among older men.

Testosterone has attracted great interest as a therapy for older men in the last few years, especially in North America^1^. Animal experiments suggest testosterone improves spatial cognition in male rats^2^,^3^. The evidence in humans is inconsistent and unclear (Table 1). Some observational studies suggest testosterone is associated with better verbal memory or higher Mini-Mental State Examination (MMSE) score^4^,^5^, although only among older men^4^ or only for MMSE at baseline^5^; whilst others suggest the association of testosterone with cognitive function is null^6^ or negative^7^. Observational studies are open to residual confounding and reverse causality^8^. Evidence from randomized controlled trials (RCTs) of testosterone administration in older men is also inconsistent, suggesting no effect^9^,^10^, or potential benefits for verbal and spatial memory^11^ or MMSE^12^. In contrast, another RCT showed a decrease in verbal memory^13^, suggesting potential harm for cognitive function. Similarly, evidence from a recent meta-analysis of interventional studies also showed unclear effects of androgen deprivation therapy on cognitive function^14^. As such, the role of testosterone in cognition remains unclear, and improved cognition remains a potential benefit^15^.

Using naturally occurring testosterone-related genetic variants in a Mendelian randomization (MR) study design provides a means of examining the causal effects of testosterone on cognitive function without any interventions. Genetic variants determined at conception, resulting in life-long differences in exposures, are unlikely to be associated with socioeconomic position or other confounders. Testosterone falls with age and ill-health^16^,^17^, and cognitive impairment has a long pre-clinical course, thus lifetime testosterone exposure may be more relevant than contemporaneous exposure in older men, however obtaining biomaterials indicating lifetime testosterone exposure is not currently feasible. An MR study with a separate-sample instrumental variable (SSIV) analysis using genetic instruments enables examination of the association of endogenous testosterone, unaffected by aging or ill-health, with cognitive function. Here, we used a separate-sample MR study design to assess the effect of endogenous peak testosterone on cognitive function in older men.

## Results

Genetically predicted log testosterone was calculated as −0.07 × rs1008805 + 0.07 × rs10046 −0.07 × rs1256031 + 3.0, as previously^18^. The proportion of the variance in log testosterone explained by genetically predicted log testosterone was 4.1%, which is typical for MR studies^19^. An instrument explaining all the variance in serum testosterone would be invalid because it would be equivalent to serum testosterone, and so would not provide an unconfounded estimate. The F-statistic was 13.3, suggesting a reliable instrument.

Among the 8,450 men in all 3 phases of the Guangzhou Biobank Cohort Study (GBCS), DNA was extracted from blood samples and used for single nucleotide polymorphism (SNP) testing for 4,262 men, with availability depending on phase of recruitment and other logistical concerns, but not on genetic profile or cognitive function. Men with SNPs tested had higher baseline delayed 10-word recall score (mean difference 0.22, 95% confidence interval (CI) 0.14–0.30) than men without SNP testing, but were not different in baseline MMSE (mean difference −0.17, 95% CI −0.37–0.03), assessed from linear regression adjusted for age; small differences could occur by chance. Among these 4,262 men, 4,212 (98.8%) had complete information on the three SNPs. None of these SNPs deviated from Hardy-Weinburg equilibrium in GBCS. Among the 4,212 men, 4,160 had 10-word recall from baseline or follow-up (3,330 from both baseline and follow-up, 712 from baseline only and 118 from follow-up only). 4,122 had MMSE score from baseline or follow-up (891 from both baseline and follow-up, 628 from baseline only, and 2,603 from follow-up only). As would be expected, genetically predicted testosterone was not associated with age, education, smoking status or use of alcohol (Table 2).

Table 3 shows genetically predicted testosterone was not associated with delayed 10-word recall score. The estimates were close to the null. The estimate for the association of genetically predicted testosterone with MMSE score was positive but the 95% CI included the null. The estimates remained unchanged after adjustment for covariates (Models 2–5) and after excluding men selected for another cognition-related project (Model 6)^20^, although in model 3 genetically predicted testosterone was associated with higher MMSE score. The association of genetically predicted testosterone with delayed 10-word recall and MMSE did not vary with age (p-values for interaction 0.13 and 0.11).

## Discussion

Using an MR study design to minimize confounding and reverse causality, we found no clear indication that endogenous testosterone was associated with improved cognitive function in older Chinese men, although we cannot rule out a small positive effect on MMSE. Our novel study provides limited support for any protective effects of endogenous testosterone on cognitive function.

The strengths of our study include using an MR study design with two separate samples (SSIV) to achieve an unbiased estimate. Simulations have shown MR with two separate samples is preferable to using the same sample for assessing the relations of the instrument with the exposure and the instrument with the outcome^21^. SSIV is useful and cost-efficient when the phenotype of interest was either not measured or was measured with substantial error in the sample with the outcome^22^,^23^. The MR design makes it feasible to use testosterone in early adulthood as a marker of lifetime exposure, when it is infeasible to obtain lifetime testosterone for older men. Thus, we avoid the imprecision in MR estimates that could arise from assessing the genetic association with testosterone in older age, when testosterone may reflect ill-health, inducing an underestimation of the genetic association with testosterone and inflating the MR estimates^24^. SSIV also remediates weak-instruments bias, reducing concerns about using multiple polymorphisms as instrument^22^. Any correlation of the genetic variants with unmeasured confounders in the sample with the phenotype is unlikely to be replicated in the sample with the outcome^22^. We also restricted the sample of young men to those with both parents and at least 3 grandparents born in Hong Kong or Guangdong province, to ensure genetic homogeneity, reflected by the similar allele frequencies of the genetic variants in the two samples^18^.

Although we used MR which can mimic the randomized treatment allocation in RCTs^25^, limitations exist. First, our SNP selection was limited by the lack of evidence from any genome-wide association study (GWAS) of testosterone in young Chinese men. Most GWAS of testosterone have been conducted in Caucasians^26^,^27^, but the SNPs identified have not all replicated successfully in Chinese men^28^. Moreover, one SNP that did replicate (rs2075230) in Chinese men is not suitable as a genetic instrument because of a pleiotropic association with sex hormone-binding globulin (SHBG)^28^. The GWAS are also conducted among older men, so the genetic associations might reflect accumulated age-related co-morbidities rather than testosterone^29^. We used a candidate gene approach rather than risk alleles from GWAS, given the relatively small sample of young men in Hong Kong, however, the requirement for a genetic instrument is to predict the exposure which may be based on functionality as well as statistical grounds^30^. The association of genetically predicted testosterone with lower high-density lipoprotein (HDL)-cholesterol^18^, as would be expected from meta-analysis of RCTs^31^, also suggests some validity of our genetic instrument. Second, the delayed 10-word recall test and MMSE might not be completely comprehensive. However, these standard tools have been validated as feasible, sensitive tools for screening mild cognitive impairment in population-based research in developing countries^32^,^33^, are widely used in epidemiological studies^34^ and have been previously used in this study^19^,^35^,^36^. Third, population stratification and canalization may affect MR. However, the participants were restricted to genetically homogeneous Chinese men. Moreover, it is generally assumed that a gene (and hence) a genetic score has the same effect on the phenotype across time and space^37^. No evidence suggests the association of the selected SNPs with testosterone varied by environmental factors. Fourth, MR is based on the assumption that the genetic instrument is associated with the exposure, is not associated with the outcome other than via the exposure (no pleiotropy) and no confounders of the association of the genetic instrument with the outcomes exist^25^. The selected SNPs would not be expected to affect cognitive function directly (pleiotropy) other than via testosterone, because the SNPs are from genes functionally relevant to testosterone. Some associations of genetically predicted testosterone with systemic inflammation, cardiovascular disease risk factors and electrocardiographic QT interval duration have been found in our previous MR studies^18^,^38^,^39^. Cardiovascular disease is associated with cognitive decline^40^, as such the association with cognitive function might be secondary to any associations with cardiovascular disease risk factors. However, additional adjustment for the Framingham score (Model 5) did not change the estimates. The genetic score was not associated with estradiol (data not shown) or the confounders (as shown in Table 2). Fifth, MR requires a large sample size. For the 10-word recall and MMSE scores, a sample size of ~4,000 has 0.8 power to detect a relatively small effect size of 0.22^41^, according to the formula for instrumental variable analysis sample size calculation given by Freeman *et al.*^41^, and consistent with simulation results given by Pierce *et al.*^42^. The null associations with delayed 10-word recall (−0.02, 95% CI −0.06–0.02) or MMSE (0.06, 95% CI −0.002–0.12) might be due to lack of power. However, the small estimates suggest little, if any, clinical benefits, for diseases, such as Alzheimer’s disease (AD), given the rapid cognitive decline with disease progression. For example, delayed 10-word recall score declines with time at a slope of 0.12 in preclinical AD^43^ and MMSE score declines at the average annual rate of 3.3 in AD patients^44^. As such, although we cannot confirm the role of testosterone, our findings which are consistent with evidence from RCTs^9^,^10^, raise doubts about the observed beneficial association of testosterone with cognitive function^4^,^5^, with relevance to those who might be attracted globally by commercial promotion of testosterone^45^. Sixth, MR assumes linearity^41^, we obtained similar results assuming a log-linear association. Seventh, the result shows no clear association of genetically predicted testosterone with cognitive function, but might suggest a potential benefit for MMSE. One possible explanation is that MMSE reflects a comprehensive measure of cognitive function whilst 10-word recall reflects verbal memory. The positive association for MMSE might reflect a potential benefit of testosterone for a specific domain of cognition, such as spatial ability, despite only a small estimate for MMSE. Replication using a specific measure of spatial cognition would be desirable. Finally, correction for multiple testing might be appropriate when using the same study for a series of outcomes. Such correction is used in agnostic or exploratory studies, such as GWAS, to control for type 1 error (false positive)^46^. However, it may increase the risk of type 2 error (false negative)^47^,^48^. Given that our study aims at confirmation and our negative findings do not corroborate observed protective associations of testosterone with cognitive function among men, controlling for the type 2 error is important from a public health perspective.

Our study is inconsistent with some observational studies where testosterone is positively or negatively associated with cognitive function^4^,^5^,^7^. However, these studies cannot distinguish whether testosterone is associated with cognitive function as a cause, as a symptom of disease or as a marker of an underlying process affecting testosterone and cognitive function. As expected, our study is consistent with some small RCTs^9^,^10^, where testosterone therapy had no effect on cognitive function in older men, although intervention in late adulthood may not be the same as lifetime intervention. The null association in our study is also consistent with observations from “lay epidemiology”^49^ where no obvious difference in overall cognitive ability exists between men and women despite very different levels of testosterone, although slight differences might exist in some specific domains, such as greater spatial ability in men^50^.

Our novel study provides a feasible and less biased method to test the lifetime effect of testosterone on cognitive function in a short time frame without any intervention. Our study adds to the limited evidence on the role of testosterone in cognitive function, providing minimal support for a protective effect of endogenous testosterone. Given emerging concerns about the use of testosterone^51^, it is unclear that the potential cognitive benefit would outweigh the risks.

## Methods

### Ethics, consent and permissions

The methods were carried out in accordance with the approved guidelines. The protocols were approved by the University of Hong Kong-Hospital Authority Hong Kong West Cluster Joint Institutional Review Board. The Guangzhou Medical Ethics Committee of the Chinese Medical Association approved GBCS, including the use of genetic data. Written, informed consent was obtained from all participants prior to participation.

### Study design

A separate-sample MR design was used (Supplementary Fig. S1). First, a genetic score predicting serum testosterone was developed in young Chinese men from Hong Kong (sample 1), with mean age of 21.0 years (standard deviation (SD) 1.8 years), as described previously^18^. Second, we applied the genetic prediction rule in the sample of older men from GBCS (sample 2), with the same genetic origin as sample 1, to examine the association of genetically predicted testosterone, rather than serum testosterone, with cognitive function among older Chinese men.

### Sources of data

Morning blood samples were collected from students recruited from the University of Hong Kong, restricted to those with both parents and at least three grandparents born in Hong Kong or Guangdong and not taking medication affecting hormones. Testosterone was assessed by competitive immunoassay on Vitros 3600 immunodiagnostic system (Ortho Clinical Diagnostics Inc, USA) with a detection limit of 0.17 nmol/L. The intra- and inter-assay coefficients of variation were 4.9% and 5.7% at 4.4 nmol/L, 3.2% and 3.9% at 16.3 nmol/L, and 1.8% and 3.0% at 37.5 nmol/L, respectively. DNA was extracted and analyzed at the Centre for Genomic Sciences of the University of Hong Kong for selected SNPs from *ESR1* (rs722208 and rs2175898), *CYP19A1* (rs10046 and rs1008805) and *ESR2* (rs1256030 and rs1256031) using a Mass ARRAY system (Sequenom, San Diego, California), for establishing the genetic prediction rule, following the flow chart (shown in Supplementary Fig. S2). A self-administered questionnaire was used to collect socioeconomic position and health status.

GBCS is an ongoing collaboration of Guangzhou Number 12 Hospital, the Universities of Hong Kong and Birmingham, UK^52^. Recruitment was in 3 phases. All participants were permanent residents of Guangzhou aged 50+ years and members of the “The Guangzhou Health and Happiness Association for the Respectable Elders” (GHHARE), a community social and welfare association unofficially aligned with the municipal government. Membership of GHHARE is open to older people for a monthly fee of 4 Yuan (50 US cents). About 7% of permanent Guangzhou residents aged 50+ years are members of GHHARE, of whom 11% (~10,000) enrolled for each of phases one, two and three. Inclusion criteria were that they were capable of consenting, ambulatory, and not receiving treatment modalities which if omitted may result in immediate life threatening risk, such as chemotherapy or radiotherapy for cancer, or dialysis for renal failure. Fasting blood samples were collected at recruitment in phase 3 and at follow-up for participants recruited in other phases. Samples were stored, as whole blood or as buffy coat and sera, at −80 °C for all apart from a subset of phase 3 participants whose DNA was extracted from fresh blood and stored at −80 °C. Selected SNPs were analyzed by a commercial company (Beijing CapitalBio Corporation) in Beijing using a Mass ARRAY system (Sequenom, San Diego, California).

### Cognitive function test

Cognitive function was assessed from a test battery developed for the Consortium to Establish a Registry for Alzheimer’s Disease (CERAD)^53^. In all 3 phases at baseline and follow-up we used the test of new learning ability (10-word list learning task). Four words (“arm”, “letter”, “ticket” and “grass”) were taken from the original CERAD English language test^33^. “Pole”, “shore”, “cabin” and “engine” were replaced with “corner”, “stone”, “book” and “stick” as in the adapted CERAD 10-word list learning task^33^. “Butter” and “queen” were replaced with “soy sauce” and “chairman” to be culturally appropriate. During the learning phase the 10-word list was read to the participants who were then asked to recall immediately. This was repeated 3 times giving immediate recall. After a 5-minute period of distraction, during which the interview was continued, the participants were asked to recall as many of the 10 words as possible. Delayed recall score is generally more predictive than immediate recall score and less affected by education^33^, so delayed rather than immediate recall score was used. The adapted Consortium 10-word list learning task has been validated as a culturally and educationally sensitive tool for identifying dementia in population-based research in developing countries^33^ and reported previously in this study^19^,^35^,^36^.

MMSE was used in phase 3 at baseline and follow-up for all phases. MMSE is the most commonly administered psychometric screening assessment of cognition^54^. MMSE is a general cognition test covering orientation, attention, memory, recall and language^55^, widely used as a relatively sensitive marker of cognitive impairment^54^,^56^,^57^. Three of 11 tasks in the original MMSE were amended to be more culturally appropriate. Orientation in place was adapted according to the geographical divisions of China and the screening setting to “country”, “province”, “city”, “hospital” and “floor”. In the 3-word registration and recall “table” and “penny” were replaced with “newspaper” and “train” to ensure all 3 words were frequently used 2-character Chinese words. The modified MMSE has the same scale as the original MMSE^58^ hence, the psychometric properties of the measures should be similar. The modified MMSE has been previously reported^19^,^35^,^36^.

### Exposure

The exposure was genetically predicted testosterone, estimated as the anti-log of predicted log testosterone from the genetic score. Serum testosterone was not measured in older men in GBCS. Testosterone, instead of log testosterone, was used for ease of interpretation as it gave the same pattern of results.

### Outcomes

The primary outcomes were delayed 10-word recall score (out of 10) and MMSE score (out of 30). The delayed 10-word recall score was normally distributed. The MMSE score was skewed, but the sample size is large^59^. Preliminary analysis showed similar results using logged MMSE, so the results in natural units are presented. Higher scores indicate better performance.

### Statistical analysis

In the young men, after testing for Hardy-Weinberg equilibrium and linkage disequilibrium, rs2175898 was dropped because it deviated from Hardy-Weinberg equilibrium, rs1256030 was dropped because it was in linkage disequilibrium with rs1256031^18^. Stepwise linear regression with all the remaining candidate SNPs (rs722208, rs10046, rs1008805, rs1256031) was used to find a parsimonious set of SNPs which best predicted log testosterone, as described previously^18^. The F-statistic, calculated from the regression of log testosterone on genetically predicted log testosterone, was obtained; an F-statistic >10 suggests a reliable genetic instrument^60^.

In the older men from GBCS, a test for trend was used to compare genetically predicted testosterone by key characteristics. We used generalized estimating equation models to estimate the relation of genetically predicted testosterone with cognition using data from both baseline and follow-up to take into account any correlation between two measurements for the same man. Although the association should not be confounded by socioeconomic position, lifestyle, or health attributes, covariates were included to achieve more precise estimates^61^. Model 1 had no covariates. Model 2 adjusted for age. Model 3 additionally adjusted for education, smoking and use of alcohol. Model 4 additionally adjusted for body mass index (BMI). Model 5 additionally adjusted for the Framingham score, because it is associated with cognitive decline^62^. We assessed whether the association of genetically predicted testosterone with cognition varied with age^4^, from the relevant interaction term. GBCS is a large cohort for answering many research questions; 690 men with all 3 SNPs for genetically predicted testosterone had SNP testing for another project on cognitive decline^20^. In model 6, we repeated the analysis with these men excluded. The power calculation was conducted using the formula for an instrumental variable analysis sample size given by Freeman *et al.*^41^, the sample size for such studies is approximately the usual sample size for exposure on outcome divided by the R^2^ between instrument and exposure. We also compared the power calculation with the simulation results of Pierce *et al.*^42^. All statistical analyses were conducted using Stata 10.1 (StataCorp LP, College Station, Texas).

## Additional Information

**How to cite this article**: Zhao, J. V. *et al.* A Mendelian randomization study of testosterone and cognition in men. *Sci. Rep.* **6**, 21306; doi: 10.1038/srep21306 (2016).

## Supplementary Material

Supplementary Information

## Figures and Tables

**Table 1:** Main findings from epidemiological studies on testosterone and cognitive function in men.

			Main references			
Study design	Exposure	Cognitivefunction	Author, year	Samplesize(men)	Mean age(years)	Main findings
Observational studies	Serum testosterone	↑ in older people	Muller, 2005^4^	400	60.2	Curvilinear association with verbal memory, which disappears in age subgroups; only associated with better verbal memory in the oldest age group, no association in other age groups. No association with MMSE.
		↑↔	Hogervorst, 2010^5^	257	74.4	Optimal TT associated with better MMSE at baseline, no association with MMSE change or verbal memory
		↔	LeBlanc, 2010^63^	1602	NA	No association with modified mental state examination (3MS), which includes MMSE
		↓	Martin, 2007^7^	1195	54.3	Associated with worse verbal memory
RCTs	Testosterone administration	↔	Haren, 2005^9^	76	68.5	No effect on MMSE
		↔	Sih, 1997^10^	15	68	No effect on verbal memory
		↓↔	Maki, 2007^13^	15	73.9	Decreased short-delay verbal memory; no effect on composite verbal memory
		↑	Cherrier, 2005^11^	32	76	Increased verbal and spatial memory
		↑	Wahjoepramono, 2015^12^	44	NA	Increased MMSE
Meta-analysis of interventional studies	Androgen deprivation therapy (ADT)	↓↔	McGinty, 2014^14^	417	63.2–71.0	Prostate cancer patients receiving ADT had worse performance on visuomotor tasks compared to noncancer control groups. No effect on other cognitive domains.

TT, total testosterone; MMSE, Mini-Mental State Examination; ADT, Androgen deprivation therapy; NA, not available currently.

↑ means a positive association with or an increase in cognitive function; ↓ means a negative association with or a decrease in cognitive function; ↔ means no association or no effect.

**Table 2:** Genetically predicted testosterone by socio-demographic characteristics among 4,212 men (50+), Guangzhou Biobank Cohort Study at baseline, 2003–2008.

			Genetically predicted testosterone(nmol/L)		
Characteristic	No.	%	Mean	SD	ANOVA *p*value^a^
Age group, years	4212				0.94
50–54		8.1	18.05	1.10	
55–59		20.2	18.01	1.22	
60–64		25.4	18.01	1.24	
65–69		24.1	18.01	1.27	
70–74		16.5	17.99	1.19	
75–79		4.2	17.95	1.28	
≥80		1.5	18.15	1.52	
Education	4209				0.91
Less than primary school		2.6	18.11	1.36	
Primary school		26.9	18.01	1.22	
Junior middle school		30.0	18.01	1.19	
Senior middle school		24.2	18.01	1.23	
Junior college		8.9	17.99	1.34	
College		7.4	17.96	1.24	
Smoking status	4192				0.59
Never smoker		40.8	17.99	1.22	
Ex-smoker		27.9	18.02	1.25	
Current smoker		31.3	18.03	1.22	
Use of alcohol	4212				0.49
Never		51.8	18.02	1.25	
<1/month		19.9	18.03	1.19	
<1/week		4.3	18.04	1.20	
1–4/week		6.0	18.03	1.30	
5+/week		11.9	17.90	1.19	
Ex-drinker		4.4	18.01	1.20	
Unknown		1.8	18.08	1.27	

Abbreviation: SD, standard deviation.

^a^Genetically predicted testosterone was not associated with age, socioeconomic position (education) or lifestyle, including smoking status and use of alcohol (ANOVA *p* value > 0.05).

**Table 3:** Effect of genetically predicted testosterone (nmol/L) on cognitive function among men (50+ years), Guangzhou Biobank Cohort Study, recruitment 2003–2008 and follow up till Dec 31, 2012.

Outcome	Mean(SD)	n	Model	Beta-coefficient^a^	95% CI	*P* value
Delayed 10-word	5.5	4160	1	−0.02	−0.06–0.02	0.23
10-word recall	(1.9)	4160	2	−0.03	−0.07–0.01	0.17
		4129	3	−0.02	−0.06–0.02	0.27
		4123	4	−0.02	−0.06–0.02	0.30
		4093	5	−0.02	−0.06–0.02	0.30
		3470	6	−0.02	−0.06–0.02	0.30
MMSE	27.5	4122	1	0.06	−0.002–0.12	0.06
	(2.7)	4122	2	0.05	−0.007–0.11	0.08
		4083	3	0.06	0.0003–0.12	0.05
		4078	4	0.06	−0.0004–0.12	0.05
		4048	5	0.06	−0.001–0.12	0.06
		3443	6	0.06	−0.01–0.13	0.10

Abbreviations: SD, standard deviation; CI, confidence interval.

Model 1 showed the association of genetically predicted testosterone with cognitive function without any covariates;

Model 2 adjusted for age;

Model 3 additionally adjusted for education, smoking, and use of alcohol;

Model 4 additionally adjusted for body mass index (BMI);

Model 5 additionally adjusted for Framingham score.

Model 6 replicated the analysis in Model 1 excluding men in another cognition project.

^a^Beta coefficient refers to the average change in cognitive function (the delayed 10-word recall score and MMSE score) with each unit (nmol/L) increase in genetically predicted testosterone.
