# Supplementary material for: A Mendelian randomization study of testosterone and cognition in men
Source: Sci Rep. 2016 Feb 11;6:21306. doi: 10.1038/srep21306 (PMC4749999; doi:10.1038/srep21306)

Title: A Mendelian randomization study of testosterone and cognition in men

Jie Zhao,[1] Tai Hing Lam,[1] Chaoqiang Jiang,[2*] Stacey S Cherny,[3] Bin Liu,[2] Kar Keung Cheng,[4] Weisen Zhang,[2] Gabriel M Leung,[1] C Mary Schooling [1,5]

Supplementary figure legends:

Supplementary Figure S1. Separate sample instrumental variable (SSIV) analysis. Sample 1 refers to young Chinese men from Hong Kong; sample 2 refers to older men from the Guangzhou Biobank Cohort Study (GBCS).

Supplementary Figure S2. Flow chart for establishing the genetic prediction rule. References 1-5 are: (1) Olson, S.H., Bandera, E.V., Orlow, I. *Am J Epidemiol*. 165, 235-45 (2007); (2) Travis, R.C., et al. *Cancer Epidemiol Biomarkers Prev.* 18, 2734-44 (2009); (3) Sowers, M.R., Wilson, A.L., Kardia, S.R., Chu, J., Ferrell, R. *Am J Med.* 119, S23-30 (2006); (4) Ahn, J., et al. *Hum Mol Genet*. 18, 3749-57 (2009); (5) Chen, Y.C., et al. *Cancer Epidemiol Biomarkers Prev.* 16, 1973-81(2007).

Supplementary Figure S1.

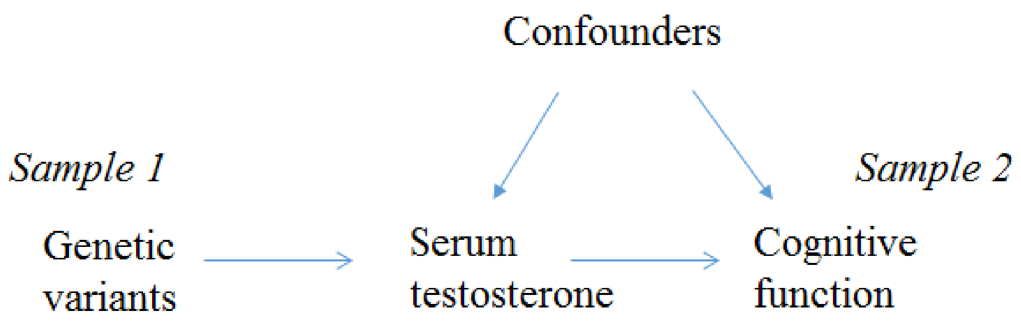

Supplementary Figure S2.

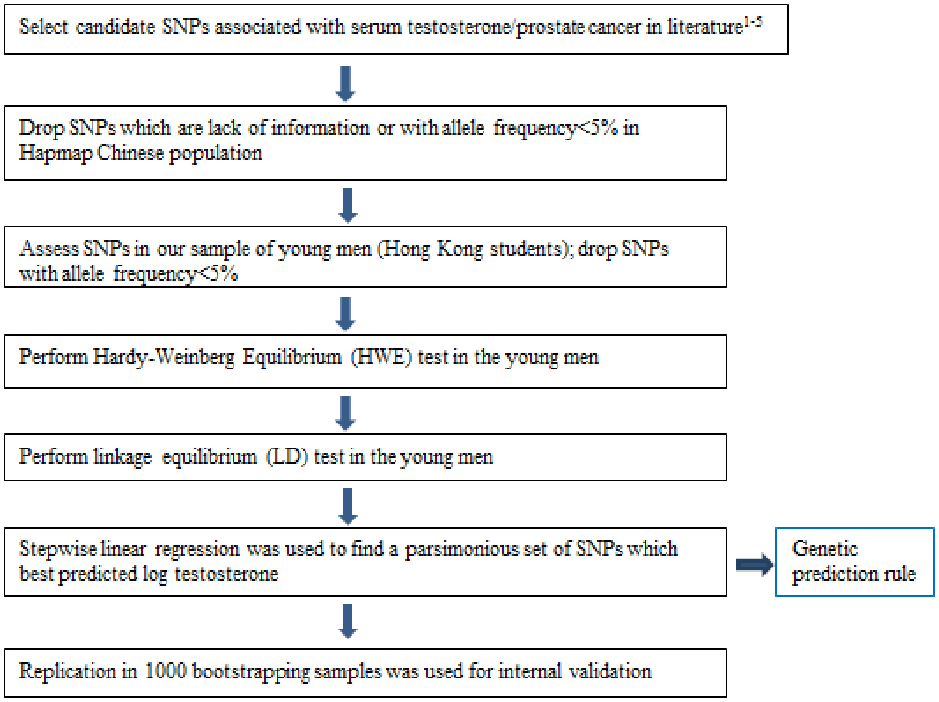

Supplement: Supplementary Information [file srep21306-s1.pdf]
